# Evaluating the implementation strategy for estimated glomerular filtration rate reporting in Manitoba: the effect on referral numbers, wait times, and appropriateness of consults

**DOI:** 10.1186/2054-3581-1-9

**Published:** 2014-05-22

**Authors:** Jay Hingwala, Sandip Bhangoo, Brett Hiebert, Manish M Sood, Claudio Rigatto, Navdeep Tangri, Paul Komenda

**Affiliations:** University of Toronto, Toronto, ON Canada; Department of Medicine, Section of Nephrology, University of Manitoba, Winnipeg, MB Canada; Seven Oaks General Hospital Renal Program, 2300 Mcphillips Street, 2PD12, Winnipeg, R2V 3M3 MB Canada; St. Boniface General Hospital, Winnipeg, MB Canada; Ottawa Hospital Research Institute, University of Ottawa, Ottawa, ON Canada

**Keywords:** eGFR, Quality improvement, Referral

## Abstract

**Background:**

Chronic kidney disease screening using estimated glomerular filtration rate (eGFR) reporting is standard in many regions. With its implementation, many centres have had higher referral rates and increased wait times to see nephrologists.

**Objective:**

Manitoba began eGFR reporting in October 2010. We measured the effect of eGFR reporting on referral rates, wait times, and appropriateness of referrals after an educational intervention.

**Design:**

An interrupted time series design was used.

**Setting:**

This study took place in Manitoba, Canada.

**Patients:**

All referrals to the Manitoba Renal Program in the period prior to eGFR reporting between April 1, 2010 and September 30, 2010 were compared with a post period between January 1, 2011 and June 30, 2011.

**Measurements:**

Data on demographics, co-morbidities, referral numbers and wait times were compared between periods. Appropriateness of consults was also measured after eGFR implementation.

**Methods:**

Prior to eGFR reporting, primary care physicians underwent educational interventions on eGFR interpretation and referral guidelines. Referral rates and wait times were compared between periods using generalized linear models. Chart audits of a random sample of 232 patients in the pre period and 239 patients in the post period were performed.

**Results:**

The pre and post eGFR reporting referral rate was 116 and 152 referrals/month, respectively. Average wait times in the pre and post eGFR reporting was 113 and 115 days, respectively. Non-urgent referral wait times increased by 40 days immediately post reporting, while urgent median referral wait times had a more gradual increase. Despite our intervention, inappropriate consultations post eGFR reporting was 495/790 (62.7%).

**Limitations:**

Our study did not measure the intervention’s success on primary care providers, which may have affected our appropriateness data. Our time series design was not powered to find a statistically significant difference in referral numbers. Residual confounding of our results was possible given the retrospective nature of our study.

**Conclusion:**

Despite our educational intervention, the inappropriate referrals remained high, and wait times increased. Other systemic interventions should be considered to attenuate the potential negative effects of eGFR reporting and ensure timely access for patients needing specialist consultation.

## Background

Chronic Kidney Disease (CKD) is a major public health problem associated with a significant burden of morbidity, mortality and increased health care costs. Early detection of CKD may help mitigate poor outcomes and reduce the high costs associated with renal replacement therapies. Accurate assessment of kidney function, also known as glomerular filtration rate (GFR), is an important part of CKD screening as it facilitates earlier diagnosis, staging, proper dosing of medications, and ultimately planning for renal replacement therapy [1]. By earlier referral to nephrology teams, a potential opportunity to attenuate CKD progression and manage complications is created [2]. GFR can be estimated (eGFR) in numerous ways, with one of the most common methods being the MDRD equation using variables of serum creatinine, age, sex, and race. Routine reporting of eGFR using the MDRD or the CKD-EPI study equation is now common in many countries and in most Canadian provinces, despite being validated only in certain populations [3].

The widespread use of automatic eGFR reporting has been shown to allow better detection of CKD in the general population [4–6], but has been associated with increased referrals to nephrologists and subsequently longer wait times [6–9]. Automatic eGFR reporting has also increased the number of inappropriate referrals to nephrologists [4]. Strategies to educate primary care clinicians in order to increase the proportion of appropriate referrals, including how to interpret eGFR’s and when to refer patients to nephrologists have been tried, but have had limited success [5].

The purpose of this study was to evaluate a multi-faceted public health campaign in Manitoba to improve the appropriateness of nephrology referrals after the implementation of province wide automatic eGFR reporting.

## Methods

### Population studied

Manitoba is a province located in central Canada, with the population of 1.27 million people with the second highest incidence and prevalence of end stage renal disease (ESRD) in Canada [10]. Over half of the population lives in the province’s capital city Winnipeg, with the remainder spread across an area of 649,950 km [2, 11]. Manitoba is culturally diverse with almost 10% of the population a member of a visible minority, and 14% aboriginal [12]. In Winnipeg, there are three dialysis centres, which together see all nephrology referrals within the province to the Manitoba Renal Program (MRP). Referrals are currently sent to a specific site for either a specific physician or into a general pool to distribute among physicians at the site. Once a referral is received, they are triaged as urgent or non-urgent by the individual nephrologist.

### Collection and reporting

The University of Manitoba Regional Ethics Board approved this study prior to its commencement. Manitoba implemented routine eGFR reporting in October of 2010. All new referrals between April 1, 2010 and September 30, 2010 were considered in this study as a sample of the pre implementation consults received to the MRP. A 3-month lag period was then observed as a transition period to the new reporting system. The post implementation sample was taken from January 1, 2011 to June 30, 2011. Thirteen weeks were chosen at random in the pre and post period representing a total of 232 patients from all three centres in the pre period and 239 patients in the post period. This sample of patients underwent complete chart reviews for demographics, co-morbidities, referral reasons, data sent with referral and wait times between referral and date seen by a nephrologist. An observed lag time of 2 years was given for primary care physicians to review and begin utilizing the MRP referral pathways before the appropriateness data was collected from a random population.

The province contains laboratory services that are both provincially and privately run. Those provincially run labs began reporting on October 25, 2010. Private labs started reporting December 31, 2010. eGFR was only reported for outpatients (ie. not in ER and inpatients). Prior to reporting, each lab was provided with IDMS traceable reference standards in order to ensure accuracy of measurement and calibration. For all patients with eGFR values greater than 60, values were standardized to be reported as “eGFR > 60” without displaying the eGFR. For those who had eGFR less than 60, the value was given along with the creatinine, with a laboratory prompt indicating that their patient may have CKD and referring them to a website containing the MRP referral guidelines and pathways (http://www.kidneyhealth.ca). EGFR was not calculated in the pre period, as it previously has been shown that without standardization to IDMS-traceable creatinine in all labs, can lead to error in measurement of eGFR [13].

### Appropriateness of referrals

A referral was deemed appropriate if any one of the following were present: 1) eGFR at time of referral of less than 30 ml/min 2) urine protein:creatinine ratio or urine albumin:creatinine ratio greater than 200 mg/mmol 3) 24 hour urine collection with greater than 2 grams protein 4) a rapid decline in eGFR of more than 10% per year or 20% in a shorter interval 5) suspected glomerulonephritis 6) structural kidney disease including renal cysts 7) special reason given by primary care physician including diabetic nephropathy, difficult to control hypertension, electrolyte abnormalities, hematuria NYD, renal stones, abnormal monoclonal deposition disease (ex. amyloid, light chain, myeloma). These criteria were clearly indicated on the referral forms at http://www.kidneyhealth.ca (an educational website maintained by the Manitoba Renal Program).

### Educational intervention

Three months prior to the eGFR reporting, the project lead nephrologist met with health administrators, lab technicians, and family physicians. Referral pathways were created in collaboration between a group of seven nephrologists and seven family physicians over several working group sessions supervised and facilitated by Manitoba Health. Each primary care physician in the province was then mailed an education package, including pathways of when to refer to nephrologists, and explanations of treatment and intervention pathways, and a poster was provided explaining the stages of CKD with coinciding therapeutic goals. Several didactic lectures took place in this period in both rural and urban sites, with lectures available by tele-link to over 8 sites in an attempt to promote and facilitate education on eGFR. In addition, a new website was launched with materials explaining how to interpret eGFR and proteinuria, chronic kidney disease stages and management, and referral pathways. Additional materials were supplemented using YouTube© videos, webcasts, live presentations, mailing lists, pamphlets, posters, and mailing an educational package to all primary care physicians and nurse practitioners in the province. When a referral was felt to be appropriate by a primary care clinician, they were instructed to complete a standardized referral form made available in the mailing and on the website. This form contained categories of emergent (consults to be seen within 24 hours), urgent (seen within 4 weeks), or elective (to be seen within 6 months) to be used in concert with the referral pathways. Emergent referrals required direct telephone contact with nephrologists who were available 24/7/365 to discuss the case; urgent referrals reasons included hematuria, suspected GN, non diabetic proteinuria, stable GFR < 15 without dialysis indications, GFR 15–30, or 30–59.5 with a decline of greater than 10% per year. Elective referral reasons were GFR less than 60, with significant proteinuria of an ACR or PCR more than 200 mg/mmol, or hematuria suspecting GN, or other reason that fulfill the above criteria (to be specified to clinician). Also requested with referrals were a medical history and additional investigations of urinalysis, proteinuria/albuminuria quantification, two eGFR measurements, CBC, electrolytes, urea, creatinine, albumin, serum and urine protein electrophoresis where appropriate, and renal ultrasound.

All materials presented to primary care physicians, including the referral pathway, are available online at http://www.kidneyhealth.ca/wp/healthcare-professionals. 6 months after the eGFR reporting, the information package was again mailed to all primary care clinicians.

### Data analysis

**Figure 1:**
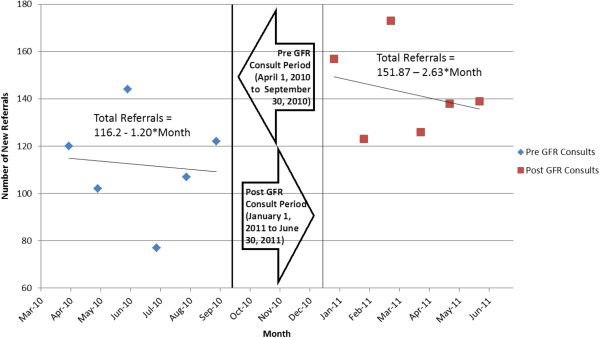
**Total new referrals by pre and post eGFR consult periods.**

**Figure 2:**
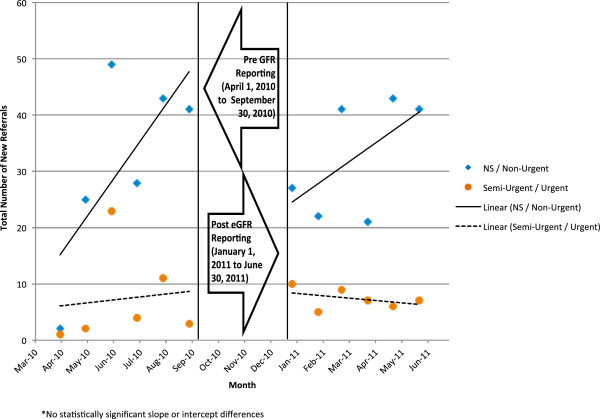
**Total new referrals by pre and post eGFR consult periods - stratified by urgency.**

**Figure 3:**
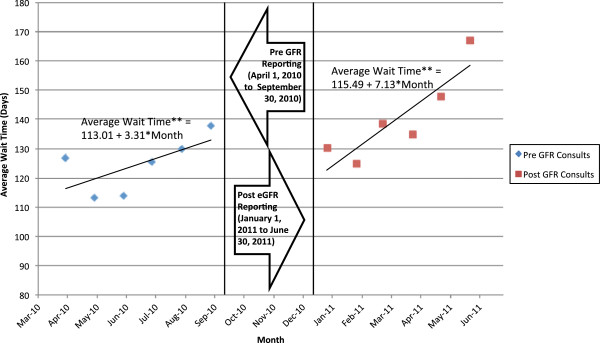
**Wait times by pre and post eGFR consult periods.** *No statistically significant difference in slope or intercept (p=0.08 & p=0.77); Intercept is defined as March 2010 for Pre GFR line of best fit, and December 2010 for Post GFR line of best fit. **Average wait time was calculated by taking weighted average weight time from four locations.

**Figure 4:**
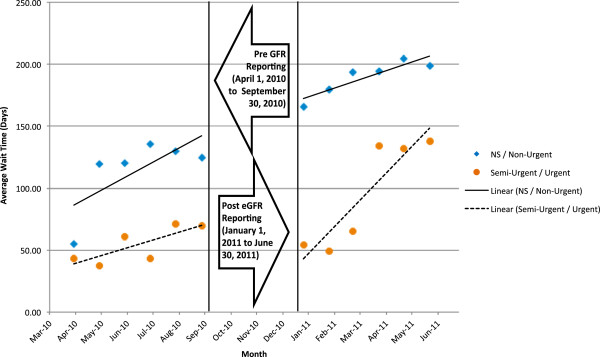
**Wait times by pre and post eGFR consult periods - stratified by urgency.** *Statisticallu significant intercept differences between Pre and Post GFR Periods for NS/Non Urgent Group (p<0.001). **Statisticallu significant slope differences between Pre and Post GFR Periods for Semi-Urgent/Urgent Group (p<0.001).

The average wait time and number of consults were compared between the pre- and post-eGFR reporting period using a general linear model. Each model contained a variable for reporting month, an indicator variable representing the post-eGFR period, and an interaction term between the reporting month and post-eGFR indicator. The immediate change in number of consults or average wait time resulting from eGFR reporting was represented by the coefficient of the post-eGFR indicator variable. The change in slope following eGFR reporting was represented by the coefficient of the interaction term between reporting month and the eGFR indicator variable. A three-month period immediately following implementation of eGFR reporting was excluded from the final analysis to allow for an appropriate transition period. Demographic comparisons were made between the pre- and post-eGFR reporting period. Continuous variables are expressed as mean and standard deviation and compared between both groups using independent t-tests. Categorical variables are expressed frequencies and percentages and compared between both groups using a chi-square test. All statistical analysis was performed using SAS version 9.2 (Cary, NC) for Microsoft Windows. Visual representations of these models (Figures 1, 2, 3 and 4) were developed using Microsoft Excel 2010 (Seattle, WA).

## Results

The patient demographics pre and post implementation of automatic eGFR reporting are shown in Table 1. The majority of patients came from Site 1, which is also the largest referral site in the province. Although not significantly different, the post implementation group did have an older mean age of 60.5. There was a similar distribution of males and females between periods. Comorbid data showed similar rates of patients with diabetes or hypertension in the pre and post periods. The group in the post period did have a larger proportion using renin-angiotensin blocking medications.

**Table 1:** **Baseline patient demographics**

Variable	Pre GFR consult		Post GFR consult		P-value
	(N=232)		(N=239)		
Age	57.3	15.8	60.5	15.4	0.0532
Referral site					0.0523
HSC	99	42.7%	139	58.2%	
SBGH	49	21.1%	57	23.9%	
SOGH	55	23.7%	43	18.0%	
Not specified	29	12.5%	0	0.0%	
Gender					0.6742
Male	127	54.7%	136	56.9%	
Female	104	44.8%	103	43.1%	
Not specified	1	0.4%	0	0.0%	
Diabetes					0.973
No	125	53.8%	122	51.1%	
Type I	8	3.5%	8	3.4%	
Type II	97	41.8%	99	41.4%	
Not Specified	2	0.9%	10	4.2%	
Hypertension					0.1062
No	83	35.8%	68	28.5%	
Yes	147	63.4%	166	69.5%	
Not specified	2	0.9%	5	2.1%	
ACE/ARB inhibitors					0.0011
No	134	57.8%	91	38.1%	
ACE only	56	24.1%	75	31.4%	
ARB only	34	14.7%	52	21.8%	
Both	7	3.0%	12	5.0%	
Not specified	1	0.4%	9	3.8%	

Categorical variables are expressed as N (%).

Numerical variables are expressed as Mean (Standard Deviation).

### Referral rates

The number of referrals received in the period before automatic eGFR reporting was about 116/month, with 109 referrals/month at the end of the pre-eGFR reporting period. In the post period, that number increased to 152 referrals/month, with 136 referrals/month at the end of this period (Figure 1). Based on our random sample of all referrals, the majority of the referrals in both periods were non-urgent (Figure 2). In the pre-eGFR period, nephrologists had a total of 672 new consult visits. In the post-eGFR period, nephrologists had 871 new consult visits. There were similar equivalent full time nephrologists working in both periods.

### Wait times

The average wait time before the eGFR period was 113 days, increasing to 115 days post implementation (Figure 3). However, the wait time’s trend continued to rise over time in the post period. Examining only non-urgent referrals, the wait time increased by 40 days immediately after GFR reporting (p < 0.01) (Figure 4). For urgent referrals, the wait time did not increase initially, but as time went on, and the overall number of referrals increased, the wait time for urgent referrals also began to rise. By the end of the six-month post-GFR period, the median wait time was 150 days for urgent referrals compared to 70 days at the end of the pre-GFR period.

### Appropriateness of referrals

In the period of January 1, 2012 to May 31, 2013, 790 random referrals were audited for appropriateness. 495/790 (62.7%) of the referrals were not considered appropriate for nephrology using our criteria. Of the appropriate referrals, 186 (23.6%) had eGFR less than 30 ml/min, 25 (3.2%) had significant proteinuria, 21 (2.7%) had both low eGFR and significant proteinuria, and 63 (8%) were referred for one of the specific reasons listed above. Of the inappropriate referrals, 386/495 (78%) stated referral reasons as a decreased eGFR. In the post period, the average patient eGFR at referral time was 40.3. The mean creatinine provided at time of referral in the pre-period was 172.3 umol/L, while the mean creatinine at referral time in the post period was lower at 139.8 umol/L.

## Discussion

The implementation of automatic eGFR reporting in Manitoba led to an increased number of nephrology referrals in the province. This resulted in a modest increased wait time for non-urgent referrals, and a concerning trend for increasing wait times in urgent referrals. Our findings parallel those of other centers, where eGFR led to more referrals, especially with older patients [4, 7, 8]. The proportion of inappropriate consultations remained high at 62.7% after our multifaceted educational intervention for primary care practitioners.

To our knowledge, only one previous study by *Phillips et al.* has simultaneously studied the combination of nephrology referrals, appropriateness of consultations, and wait times in the context of automatic eGFR reporting [14]. This study was conducted in the United Kingdom, where a significant proportion of income for primary care physicians are linked to certain quality targets in CKD management. After eGFR reporting began, a similar rise in nephrology consultations was reported. Shortly after, a patient referral pathway to guide primary care physicians for appropriateness of referral was disseminated, and found to improve the appropriateness of consults. Canada differs from the UK system in that it does not tie financial reimbursement to quality targets in primary care, and therefore a similar strategy may not be as effective in attenuating inappropriate consult rates. We therefore attempted a more robust educational strategy including an analogous referral pathway as the *Phillips et al.* study.

Automatic eGFR reporting has led to more referrals in many studies, which in many cases has allowed the capture of more patients with CKD [15, 16]. Earlier identification of these patients by primary care clinicians allows for treatment to attenuate CKD progression to ESRD through interventions such as tight blood pressure control, renin-angiotensin system inhibitors, and consultation with a nephrologist [17–20]. The STAART study demonstrated that patients who have follow up for greater than one year by a nephrologist are more likely to have optimal dialysis starts with pre-emptively created fistulas, start on peritoneal dialysis, begin dialysis as elective outpatients, and have a lower mortality rates. However, the increased referral rates that have been seen with eGFR reporting have not paralleled a significant change in “appropriate nephrology consultations” or lessened the progression to end stage renal disease. This is likely due to the fact that the majority of the new referrals are at low risk of CKD progression to ESRD [4, 5, 9].

Improving front line providers knowledge and awareness of CKD is an essential element for effective, “appropriate”, risk based triage of referrals to nephrology [21]. Primary care clinicians have indicated a need for more education in areas of eGFR interpretation, clinical utility, and methods of conveying its meaning to patients [16, 22]. They have also expressed that they would like feedback on their current CKD care practices, when to refer to a specialist, established roles in clinical care before and after nephrology referrals, and regular updates on current guidelines of best practices [16, 23]. The need to provide support in these areas is reinforced by multiple studies, which have illustrated disparities in primary care clinicians CKD knowledge translation, recognition of CKD risk factors [24], interpretation of creatinine and eGFR measurements, diagnostic evaluation for CKD, and utilization of current management guidelines [25]. Our program employed a comprehensive public health strategy to encourage proper utilization of the eGFR tool by deriving referral pathways in collaboration between nephrologists and primary care physicians and using mass mail outs, didactic presentations, web-based videos, and dedicated web-based resources to disseminate knowledge on appropriate referrals. Despite these efforts, the numbers of inappropriate referrals remained high and wait times increased.

There are several clinical, research and public health implications to our findings. Clinical practice guidelines have been a popular strategy used to address gaps in CKD recognition and advise when to prompt a nephrology referral. Similar to our study however, practice guidelines have not been an effective solution to bridge these inconsistencies. Previous studies have described reasons why guidelines are ineffective in primary care, including provider lack of awareness and familiarity with guidelines, disagreement with recommendations, lack of confidence with self-efficacy or time to carry out recommendations, apprehension to change previous practices, and absence of external pressures to change practices [26]. In the case of nephrology, this problem is compounded by regional variation of guidelines [27, 28], the increasingly complexity of making a CKD diagnosis, the development of new equations such as CKD-EPI and cystatin C, and the incorporation of proteinuria with staging [29].

Recognition of high risk patients by simpler methods of interpretation, such as printed reports with statements reminding appropriate referral guidelines, or the KDIGO heatmap [30, 31], could incorporate these new variables into a more concise model that would be easier to apply in primary care. Computer decision support systems [32, 33] such as electronic medical records [25] or practice enhancement assistants [34] are potential promising solutions in increasing physician awareness and detection of CKD, decreasing duplication of tests, ease comparisons with prior results, and providing alerts when labs results return. It also has the ability to advise drug-dosing, interpretation of results, and provide referral suggestions. One additional benefit of computer support systems for patients, is that they can review their individual results from their own computers, potentially improving their interest and self-management of their own diseases [25]. A potential effective approach to improve practice patterns would be to audit current performances of primary care providers, then convey benchmarks or comparisons of certain indicators to them while reinforcing indications for an appropriate referral to nephrology [33]. This type of feedback on current performance has been shown in numerous quality improvement initiatives to improve CKD awareness and management, especially when combined with key opinion leaders messages’ [31].

A more robust and perhaps more effective strategy may be to intervene on the process of referral intake. Centralized, standard referral processes allows for a single triage area to receive, process, screen for appropriateness of the referral and completeness of the consultation, and to evenly distribute referrals. More urgent consults could be better triaged, completeness of referral information should be ensured to prevent secondary delays, and wait lists can be balanced for each nephrologist. Subspecialty nephrology clinics, such as glomerulonephritis, hypertension, CKD with pregnancy, or genetic disease clinics, can have referrals directed toward them more efficiently as well in a triage based system. Utilization of risk prediction equations [35] can also help prioritize the urgency and necessity for low risk patients to be seen by nephrology.

This study has several strengths, including a random sample of groups in the pre and post eGFR implementation periods for group comparisons of demographics and wait times. The MRP receives all nephrology referrals in the province allowing for virtually complete data capture of referred patients. Although our educational intervention was multi-faceted, one limitation of our study was that we did not measure how well primary care providers reviewed or grasped the content, which may have affected our appropriateness data. Another limitation in our time series design is that we provide 6 data points in the pre and post periods for number of referrals, which yielded a trend, but may have been statistically significant had we had increased power. We did not report race with our demographics, as this information was not available to us. However, Manitoba has a small African American population of less than 1.4% [36], so the calculated mean eGFR was unlikely to be largely influenced by this missing information. In addition, the retrospective nature of our study did not allow us to measure all key statistics that could influence our outcomes, making residual confounding of our results a possibility.

## Conclusions

In conclusion, EGFR implementation has shown to be a useful tool in CKD screening, but with it has come challenges of increase nephrology referrals and longer wait times. In Manitoba, despite our significant efforts at a robust knowledge translation initiative we also encountered similar setbacks. A higher yield to improve the referral intake process would perhaps be to better align risk of CKD progression with resource utilization, which in turn may lead to a prospect for larger gains in improving the quality of care.

### Consent

Informed consent was waived from our regional ethics board as there was no intervention, only aggregate data was presented, and it was impractical to go back to obtain informed consent for a study deemed very low risk.

## Abbreviations

eGFR: Estimated glomerular filtration rate
CKD: Chronic kidney disease
GFR: Glomerular filtration rate
MDRD: Modification of diet in renal disease
CKD-EPI: Chronic kidney disease epidemiology
MRP: Manitoba renal program
NYD: Not yet determined
ESRD: End stage renal disease
KDIGO: Kidney disease improving global outcomes.

## References

[1] Fesler P, Mimran A (2011). Estimation of glomerular filtration rate: what are the pitfalls?. Curr Hypertens Rep.

[2] Kanda E, Erickson K, Bond TC, Krisher J, McClellan WM (2011). Hemodialysis treatment center early mortality rates for incident hemodialysis patients are associated with the quality of care prior to starting but not following onset of dialysis. Am J Nephrol.

[3] Levey AS, Bosch JP, Lewis JB, Greene T, Rogers N, Roth D (1999). A more accurate method to estimate glomerular filtration rate from serum creatinine: a new prediction equation. Modification of diet in renal disease study group. Ann Intern Med.

[4] Noble E, Johnson DW, Gray N, Hollett P, Hawley C, Campbell S, Mudge D, Isbel N (2008). The impact of automated eGFR reporting and education on nephrology service referrals. Nephrol Dial Transplant.

[5] Akbari A, Grimshaw J, Stacey D, Hogg W, Ramsay T, Cheng-Fitzpatrick M, Magner P, Bell R, Karpinski J (2012). Change in appropriate referrals to nephrologists after the introduction of automatic reporting of the estimated glomerular filtration rate. CMAJ.

[6] Jain A, Hemmelgarn BR (2011). Impact of estimated glomerular filtration rate reporting on nephrology referrals: a review of the literature. Curr Opin Nephrol Hypertens.

[7] Hemmelgarn BR, Zhang J, Manns BJ, James MT, Quinn RR, Ravani P, Klarenbach SW, Culleton BF, Krause R, Thorlacius L, Jain AK, Tonelli M (2010). Nephrology visits and health care resource use before and after reporting estimated glomerular filtration rate. JAMA.

[8] Jain AK, McLeod I, Huo C, Cuerden MS, Akbari AA, Tonelli M, van Walraven C, Quinn RR, Hemmelgarn B, Oliver MJ, Li P, Garg AX (2009). When laboratories report estimated glomerular filtration rates in addition to serum creatinines, nephrology consults increase. Kidney Int.

[9] Naimark DM, Harel Z, Moineddin R, Bergman A (2012). The impact of estimated glomerular filtration rate reporting on nephrology referral pattern, patient characteristics and outcome. Nephron Clin Pract.

[10] Canadian Institute for Health Information (2011). Treatment of End-Stage Organ Failure in Canada, 2000 to 2009.

[11] Winnipeg Regional Health Authority (2010). Community Health Assessment 2009/2010.

[12] Aboriginal Peoples in Canada (2011). First Nations People, Metis and Inuit.

[13] Komenda P, Beaulieu M, Seccombe D, Levin A (2008). Regional implementation of creatinine measurement standardization. J Am Soc Nephrol.

[14] Phillips LA, Donovan KL, Phillips AO (2009). Renal quality outcomes framework and eGFR: impact on secondary care. QJM.

[15] Akbari A, Swedko PJ, Clark HD, Hogg W, Lemelin J, Magner P, Moore L, Ooi D (2004). Detection of chronic kidney disease with laboratory reporting of estimated glomerular filtration rate and an educational program. Arch Intern Med.

[16] Smith DH, Schneider J, Thorp ML, Vupputuri S, Weiss JW, Johnson ES, Feldstein A, Petrik AF, Xuihai Yang X, Snyder SR (2012). Clinician’s use of automated reports of estimated glomerular filtration rate: a qualitative study. BMC Nephrol.

[17] Arora P, Obrador GT, Ruthazer R, Kausz AT, Meyer KB, Jenuleson CS, Pereira BJ (1999). Prevalence, predictors, and consequences of late nephrology referral at a tertiary care center. J Am Soc Nephrol.

[18] Obrador GT, Ruthazer R, Arora P, Kausz AT, Pereira BJ (1999). Prevalence of and factors associated with suboptimal care before initiation of dialysis in the United States. J Am Soc Nephrol.

[19] Jungers P, Zingraff J, Albouze G, Chauveau P, Page B, Hannedouche T, Man NK (1993). Late referral to maintenance dialysis: detrimental consequences. Nephrol Dial Transplant.

[20] Black C, Sharma P, Scotland G, McCullough K, McGurn D, Robertson L, Fluck N, MacLeod A, McNamee P, Prescott G, Smith C (2010). Early referral strategies for management of people with markers of renal disease: a systematic review of the evidence of clinical effectiveness, cost-effectiveness and economic analysis. Health Technol Assess.

[21] Boulware LE, Troll MU, Jaar BG, Myers DI, Powe NR (2006). Identification and referral of patients with progressive CKD: a national study. Am J Kidney Dis.

[22] Fox CH, Brooks A, Zayas LE, McClellan W, Murray B (2006). Primary care physicians’ knowledge and practice patterns in the treatment of chronic kidney disease: an Upstate New York Practice-based Research Network (UNYNET) study. J Am Board Fam Med.

[23] Campbell KH, Smith SG, Hemmerich J, Stankus N, Fox C, Mold JW, O’Hare AM, Chin MH, Dale W (2011). Patient and provider determinants of nephrology referral in older adults with severe chronic kidney disease: a survey of provider decision making. BMC Nephrol.

[24] Lea JP, McClellan WM, Melcher C, Gladstone E, Hostetter T (2006). CKD risk factors reported by primary care physicians: do guidelines make a difference?. Am J Kidney Dis.

[25] Shahinian VB, Saran R (2010). The role of primary care in the management of the chronic kidney disease population. Adv Chronic Kidney Dis.

[26] Cabana MD, Rand CS, Powe NR, Wu AW, Wilson MH, Abboud PC, Rubin HR (1999). Why don’t physicians follow clinical practice guidelines? A framework for improvement. JAMA.

[27] Campbell GA, Bolton WK (2011). Referral and comanagement of the patient with CKD. Adv Chronic Kidney Dis.

[28] Lopez-Vargas PA, Tong A, Sureshkumar P, Johnson DW, Craig JC (2013). Prevention, detection and management of early chronic kidney disease: a systematic review of clinical practice guidelines. Nephrology (Carlton).

[29] Matsushita K, Tonelli M, Lloyd A, Levey AS, Coresh J, Hemmelgarn BR (2012). Clinical risk implications of the CKD Epidemiology Collaboration (CKD-EPI) equation compared with the Modification of Diet in Renal Disease (MDRD) Study equation for estimated GFR. Am J Kidney Dis.

[30] Levey AS, de Jong PE, Coresh J, El Nahas M, Astor BC, Matsushita K, Gansevoort RT, Bertram L, Kasiske BL, Eckardt K (2011). The definition, classification, and prognosis of chronic kidney disease: a KDIGO Controversies Conference report. Kidney Int.

[31] Ivers N, Jamtvedt G, Flottorp S, Young JM, Odgaard-Jensen J, French SD, O'Brien MA, Johansen M, Grimshaw J, Oxman AD (2012). Audit and feedback: effects on professional practice and healthcare outcomes. Cochrane Database Syst Rev.

[32] Garg AX, Adhikari NK, McDonald H, Rosas-Arellano MP, Devereaux PJ, Beyene J, Sam J, Haynes RB (2005). Effects of computerized clinical decision support systems on practitioner performance and patient outcomes: a systematic review. JAMA.

[33] Klebe B, Farmer C, Cooley R, de Lusignan S, Middleton R, O'Donoghue D, New J, Stevens P (2007). Kidney disease management in UK primary care: guidelines, incentives and information technology. Fam Pract.

[34] **Primary care recognition and treatment of chronic kidney disease can be markedly improved with a few approaches** 2009. Accessed at http://www.ahrq.gov/news/newsletters/research-activities/jun09/0609RA8.html

[35] Rigatto C, Sood MM, Tangri N (2012). Risk prediction in chronic kidney disease: pitfalls and caveats. Curr Opin Nephrol Hypertens.

[36] *Visible minority population, by province and territory (2006 census)* Government of Canada; 2009. Accessed March 1, 2014, at http://www.statcan.gc.ca/tables-tableaux/sum-som/l01/cst01/demo52b-eng.htm

